# Computational study on new natural compound agonists of dopamine receptor

**DOI:** 10.18632/aging.203180

**Published:** 2021-06-25

**Authors:** Hui Li, Wenzhuo Yang, Jianxin Xi, Zhenhua Wang, Han Lu, Zhishan Du, Weihang Li, Bo Wu, Shanshan Jiang, Yida Peng, Jingyi liu, Luwei liu, Xiangheng Zhang, Jiachun Feng

**Affiliations:** 1 Department of Neurology, The First Hospital of Jilin University, Changchun, China; 2 Department of Neurosurgery, State Key Laboratory of Oncology in South China, Collaborative Innovation Center for Cancer Medicine, Sun Yat-Sen University Cancer Center, Guangzhou, China; 3 Clinical College, Jilin University, Changchun, China; 4 Department of Orthopaedic Surgery, Xijing Hospital, The Fourth Military Medical University, Xi'an, China; 5 Department of Orthopaedic Surgery, The First Hospital of Jilin University, Changchun, China; 6 College of Computer Science and Technology, Jilin University, Changchun, China; 7 Chinese Academy of Sciences, Research Group of Evolution and Population Genomics, Institute of Zoology, Beijing, China; 8 Department of Biomedical Informatics, Harvard Medical School, Cambridge, MA 02115, USA

**Keywords:** dopamine receptor (DAR), agonist, virtual screening, prolactinoma, Parkinson's disease

## Abstract

Dopamine receptor, a polypeptide chain composed of 7 hydrophobic transmembrane regions, is a new and vital drug target, especially Dopamine receptor 2(D2). Targeting dopamine receptors, Dopamine receptor agonists are a class of drugs similar in function and structure to dopamine and can directly act on dopamine receptors and activate it. Clinically, Dopamine receptor agonist drugs have achieved significant therapeutic effects on prolactinoma and Parkinson's Disease.

In the study, we virtually screened a series of potential effective agonists of Dopamine receptor by computer techniques. Firstly, we used the Molecular Docking (LibDock) step to screen out some molecules that can dock well with the protein. Then, analysis of toxicity prediction and ADME (adsorption, distribution, metabolism and excretion) were carried out. More precise molecular docking (CDOCKER) and 3-Dimensional Quantitative Structure-Activity Relationship Modeling Study(3D-QSAR) pharmacophore generation were implemented to research and explore these compounds' binding mechanism with Dopamine receptor. Last but not least, to assess compound's binding stabilities, we carried out a molecular dynamic analysis. As the results show, two compounds (ZINC000008860530 and ZINC000004096987) from the small molecule database (ZINC database) were potential effective agonists of Dopamine receptor. These two compounds can combine with Dopamine receptor with higher affinity and proved to be no toxic. The cell experiment showed that two compounds could inhibit the proliferation and PRL secretion of MMQ cells (pituitary tumor cells). Thus, this study provided valuable information about Dopamine receptor agonist-based drug discovery. So, this study will benefit patients with prolactinoma and Parkinson’s disease a lot.

## INTRODUCTION

Dopamine receptor (DAR), a polypeptide chain consisting of 7 hydrophobic cross-membrane regions, is a new and important drug target [1]. Targeting dopamine receptors, DAR agonists are a class of drugs similar in function and structure to dopamine and can directly act on dopamine receptors [2, 3]. Clinically, DAR agonist drugs have achieved significant therapeutic effects on many diseases, such as prolactinoma, Parkinson's Disease, etc. [4].

Additionally, DAR is a general term for a series of receptors, including d1, d2, d3, d4, d5. And according to amino acid sequence and the signal conduction coupling, DAR is divided into D1 receptors (d1 and d5 receptors subtypes) and D2 receptors (d2, d3, and d4 receptor subtypes) [5]. Furthermore, DAR agonists have different affinities for different types of dopamine receptors. And they have different reactions and effects when acting on different dopamine receptors. And different dopamine receptors have different cell signaling pathway mechanisms. First of all, DAR couples with adenylate cyclase G protein (Gs). D1 receptors, by stimulating adenylate cyclase G protein (Gs), can couple with the Adenylate cyclase positively and activate it. Then activated Adenylate cyclase can increase the level of cAMP. Nevertheless, by inhibiting adenylate cyclase G protein (Gs), D2 receptors can couple Adenylate cyclase negatively and inhibit it [6]. Consequently, the inhibition of Adenylate cyclase will reduce the level of cAMP. CAMP acts as the second messenger in the cell signal pathway, and the information is transmitted from the first messenger to the second messenger [7]. In the cell, cAMP turns the inactive protein kinases into active, thereby activating phosphorylase and then causing reactions of target cells, such as glandular cell secretion, muscle cell contraction and relaxation, nerve cell potential changes, cellular changes in permeability, cell division and differentiation, and various enzyme reactions, etc.

More importantly, DAR agonists mainly work by acting on D2 receptors [8]. And DAR agonists have been used in the clinical treatment of prolactin adenoma for a long time and have an excellent therapeutic effect on Parkinson's disease clinically [9]. Firstly, prolactinoma is the most common type of pituitary tumors, which is characterized by excessive prolactin (PRL) secretion as a neuroendocrine-related disease. Additionally, elevated prolactin levels can damage the reproductive system and sexual function through the various links of the hypothalamus-pituitary-gonadal axis. Currently, it is believed that DAR agonists can inhibit the secretion of PRL by binding to D2 DAR expressed on the surface of normal prolactin and pituitary tumor cells [10]. And the reduction of PRL secretion is achieved through the following mechanisms after DAR agonists activated D2 DAR: Within a few seconds, hyperpolarization of the cell membrane causes an increase in K + conduction, and an increase in K + conduction causes the voltage-gated Calcium channel to close, resulting in a decrease in intracellular free Ca2 +. And the release of prolactin stored in vesicles is positively correlated with free Ca2+, so the reduction of Ca2+ reduces the release of prolactin. [11]; in addition, within minutes to hours of activation of dopamine receptors, dopamine is coupled to dopamine receptors to activate Gai and Gao protein subtypes, thereby changing the activity of adenylate cyclase, which in turn reduces cAMP levels and ultimately Inhibited PKA activity. And PKA ultimately reduces the expression of the PRL gene by phosphorylation of cytoplasm and nuclear protein [12]; There is another aspect. Within a few days, dopamine receptors are activated to inhibit prolactinoma cell proliferation and reduce cell volume [13]. The third mechanism is still under discussion and needs further study.

Moreover, agonists of DAR also has an ideal therapeutic effect on Parkinson's disease by improving tremor, stiffness, slow movement, and any complications of Parkinson's Disease stage such as delay or "off" [9]. The striatum-thalamus-cortex circuit regulate human activity. And as a chronic progressive disease, Parkinson's Disease is characterized by a reduction of dopamine in dopaminergic nerve endings striatum for the degeneration of dopaminergic neurons in the substantia nigra striatum. Under normal circumstances, the dopamine pathway can be divided into direct pathway (D1 receptor participation) and indirect pathway (D2 receptor participation). When the direct pathway is excited, the human body is guaranteed to move, while when the indirect pathway is excited, it inhibits unwanted activities. The two are in a balanced state to ensure normal activities. Due to the lack of dopamine in Parkinson's disease, the effect on the direct pathway is weakened, and normal activities are reduced; the inhibitory effect on the indirect pathway is weakened, so that the indirect pathway excessively inhibits unwanted exercise activities, resulting in symptoms such as reduced exercise and muscle stiffness. Currently, DAR agonists on the market directly stimulate D2 receptors and have a weak inhibitory effect on D1 receptors so that the direct pathway and the indirect pathway return to normal or close to normal.

It can be seen that agonists of DAR have good prospects in treatment of prolactinoma and Parkinson's Disease. Therefore, DAR agonists are a potential target drug molecule [14]. Additionally, as DAR agonists are widely used in clinical practice and have sound effects, there have been many studies on DAR agonists in recent years. There are also many existing DAR agonists. According to chemical structures, DAR agonists including two categories, non-ergot derivatives (include quigolide, and etc.) and ergot derivatives (include bromocriptine, pergolide and cabergoline) [15]. Nevertheless, many existing agonist-drugs of DAR have side effects, such as nausea, vomiting, headache, dizziness, etc. And there are fewer agonists of DAR with ideal therapeutic effect. Thus, studying the pharmacological effects of DAR agonists, finding drugs with high efficiency and low side effects, and applying them more widely in clinical practice, especially for prolactinoma and Parkinson's disease, are necessary and beneficial. Among DAR agonists, Bromocriptine has been used in the clinical treatment of prolactin adenoma for the longest time and has an excellent therapeutic effect on prolactinoma and Parkinson's disease clinically [9]. Therefore, Bromocriptine was chosen as the reference molecule of DAR agonists in our study.

As reported recently, natural molecules have contributed significantly to not only molecular biological research but also potential drug development. And to find favorable DAR agonists, we performed a virtual screening based on the ZINC database [16, 17]. Then, we analyzed molecules' toxicity properties and ADME (absorption, distribution, metabolism and excretion). By docking, we also analyzed the modes how potential compounds and DAR interact and bind with each other. To assess if their bind stably, we performed a molecular dynamics simulation. Finally, a Cell Counting Kit 8 (CCK8) assay and ELISA were performed to verify the effect of potential compounds. This study provided potential inhibitor’s pharmacological properties, which will significantly promote the development of DAR agonist-drugs [18, 19].

## RESULTS

### To virtually screen potential agonists of DAR

DAR agonists are a class of drugs similar in function and structure to dopamine and can directly act on dopamine receptors. The currently marketed DAR agonists mainly act on D2 receptors. For example, bromocriptine directly stimulates D2 receptors and has a weak inhibitory effect on D1 receptors. Additionally, Bromocriptine has achieved significant clinical results in treating prolactinoma and Parkinson's disease, and it is a generally recognized representative DAR agonist. Therefore, we chose the region where bromocriptine and dopamine receptors bound and interacted to definite the combining center and range of the ball. Then the ball was used to set the parameters of the binding and interaction area in virtual screening. Bromocriptine and DAR complex was downloaded from PubMed protein database. Firstly, we performed LibDock for virtual screening to find favorable agonists of DAR by Discovery Studio 4.5 (DS4.5, Accelrys, Inc., San Diego, CA, USA). 25932 molecules that are natural and purchasable were downloaded for free from the ZINC database. The database is established by Irwin and Shoichet Laboratories in the Department of Pharmaceutical Chemistry at the University of California, San Francisco [18]. Additionally, the 3D structure of DAR was regarded as target in this study. Additionally, the well-known DAR agonists Bromocriptine was chosen Bromocriptine as the reference compound that could activate DAR activity *in vitro* and *vivo*. DAR is a polypeptide chain consisting of 7 hydrophobic cross-membrane regions which couples with adenylate cyclase G protein (Gs). By stimulating adenylate cyclase G protein (Gs), they can increase cells and the body's activities through cell signaling pathways. So, the binding area of compounds and DAR are potential therapeutic target. According to the screening results, 141 compounds’ scores of LibDock are higher than Bromocriptine (147.011). The top 20 molecules are listed in Table 1.

**Table 1:** Top 20 ranked compounds with higher LibDock scores than bromocriptine.

**Number**	**Compounds**	**LibDock score**	**Number**	**Compounds**	**LibDock score**
1	ZINC000004424205	172.991	11	ZINC000002526388	157.683
2	ZINC000045337516	170.080	12	ZINC000002528509	156.895
3	ZINC000014811803	166.753	13	ZINC000008214547	155.494
4	ZINC000100590636	165.693	14	ZINC000062238181	154.742
5	ZINC000017596232	164.541	15	ZINC000008860530	154.326
6	ZINC000015122022	161.842	16	ZINC000049180748	153.298
7	ZINC000100634117	160.977	17	ZINC000004096987	152.728
8	ZINC000002566164	160.816	18	ZINC000008221074	151.618
9	ZINC000100634116	159.633	19	ZINC000011616465	148.919
10	ZINC000004097774	158.333	20	ZINC000006920421	148.455

### Assession of pharmacological properties for ADME and toxicity

By carrying out Discovery Studio 4.5’s ADME module, we analyzed pharmacological properties of Bromocriptine and promising compounds, including brain/blood barrier (BBB), aqueous solubility, human intestinal absorption, cytochrome P450 2D6 binding (CYP2D6), hepatotoxicity and plasma protein binding properties (PPB) (Table 2) [5, 20]. As for the aqueous solubility, it was analyzed at temperature of twenty-five° C. Results showed that all molecules have good solubility other than ZINC000062238181, ZINC000008221074 and ZINC000006920421 in water. 7 compounds’ solubility was favorable while 7 compounds’ solubility was moderate. Bromocriptine had low, but possible solubility. As for the absorption of human intestinal, 5 molecules and Bromocriptine had poor level of absorption while 4 molecules had suitable level of absorption. Additionally, we found 9 compounds as well as Bromocriptine to interact and combine with plasma protein tightly, but the others was not. ZINC000015122022, ZINC000002566164, ZINC000002526388, ZINC000002528509, ZINC000062238181, ZINC000008221074 and ZINC000006920421 inhibited cytochrome P450 2D6 (CYP2D6), but the rest did not. CYP2D6 mattered a lot in drug’s metabolism [21]. Bromocriptine was proved to have none inhibition of CYP2D6, too. 15 compounds were non-hepatotoxic. And the rest were hepatotoxic.

**Table 2:** ADME (adsorption, distribution, metabolism, excretion) properties of compounds.

**Number**	**Compounds**	**Solubility level^a^**	**BBB level^b^**	**CYP2D6^c^**	**Hepatotoxicity^d^**	**Absorption level^e^**	**PPB level^f^**
1	ZINC000004424205	2	4	0	1	3	0
2	ZINC000045337516	1	4	0	0	3	1
3	ZINC000014811803	3	4	0	1	3	0
4	ZINC000100590636	3	4	0	0	2	0
5	ZINC000017596232	1	4	0	0	3	0
6	ZINC000015122022	2	4	1	0	2	1
7	ZINC000100634117	3	4	0	0	2	0
8	ZINC000002566164	2	4	1	0	3	0
9	ZINC000100634116	3	4	0	0	2	0
10	ZINC000004097774	2	4	0	0	3	0
11	ZINC000002526388	2	4	1	1	0	1
12	ZINC000002528509	2	4	1	1	0	1
13	ZINC000008214547	2	4	0	0	0	0
14	ZINC000062238181	0	4	1	0	3	1
15	ZINC000008860530	3	4	0	0	2	1
16	ZINC000049180748	3	4	0	1	3	0
17	ZINC000004096987	4	4	0	0	0	1
18	ZINC000008221074	0	4	1	0	3	1
19	ZINC000011616465	2	4	0	0	3	0
20	ZINC000006920421	0	4	1	0	3	1
21	Bromocriptine	1	4	0	1	2	1

^a^Aqueous-solubility level: 0 (extremely low); 1 (very low, but possible); 2 (low); 3 (good).

^b^Blood Brain Barrier level: 0 (Very high penetrant); 1 (High); 2 (Medium); 3 (Low); 4 (Undefined).

^c^Cytochrome P450 2D6 level: 0 (Non-inhibitor); 1 (Inhibitor).

^d^Hepatotoxicity: 0 (Nontoxic); 1 (Toxic).

^e^Human-intestinal absorption level: 0 (good); 1 (moderate); 2 (poor); 3 (very poor).

^f^Plasma Protein Binding: 0 (Absorbent weak); 1 (Absorbent strong).

**Figure 1:**
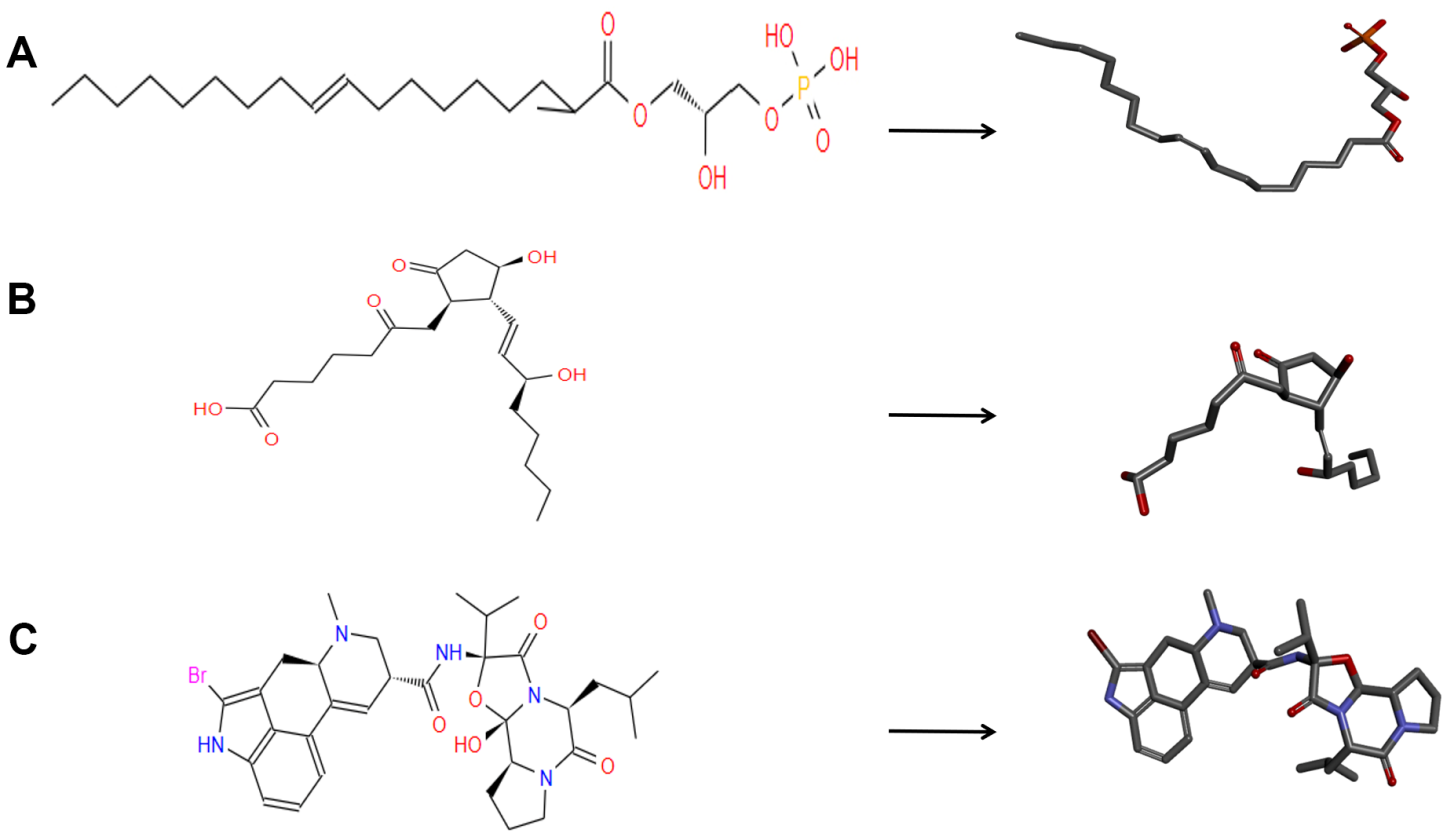
**The 2D structures of bromocriptine and novel compounds selected from virtual screening by chemdraw.** And 3D structures of Bromocriptine and novel compounds selected from virtual screening by DS 4.5. (**A**) ZINC000008860530; (**B**) ZINC000004096987; (**C**) Bromocriptine.

**Figure 2:**
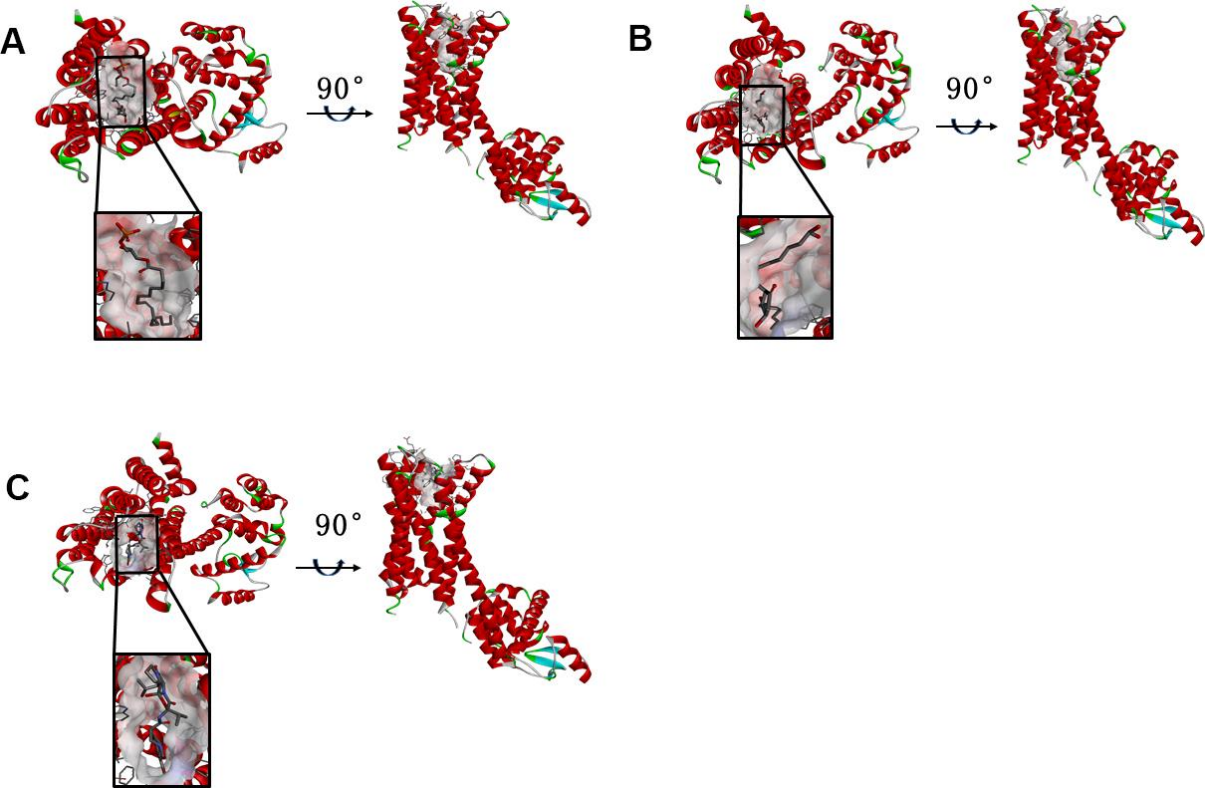
(**A**) ZINC000008860530-Dopamine D2 Receptor complex. Schematic drawing of interactions between ligands and Dopamine D2 Receptor, and the Ionizability surface of the junction pocket was added, blue represented basic ionization, red represented acid ionization, and ligands were shown in sticks, the structure around the ligand-receptor junction were shown in thinner sticks. (**B**) ZINC000004096987-Dopamine D2 Receptor complex. Schematic drawing of interactions between ligands and Dopamine D2 Receptor, and the Ionizability surface of the junction pocket was added, blue represented basic ionization, red represented acid ionization, and ligands were shown in sticks, the structure around the ligand-receptor junction were shown in thinner sticks. (**C**) Bromocriptine-Dopamine D2 Receptor complex. Schematic drawing of interactions between ligands and Dopamine D2 Receptor, and the Ionizability surface of the junction pocket were added, blue represented basic ionization, red represented acid ionization, and ligands were shown in sticks, the structure around the ligand-receptor junction were shown in thinner sticks.

Bromocriptine was hepatotoxic. Afterwards, safety properties of the top 20 ranked compounds and Bromocriptine, including AMES (Ames mutagenicity), DTP (developmental toxicity potential) and Rodent carcinogenicity were analyzed by Discovery Studio 4.5’s TOPKAT module. In addition, the carcinogenicity of rodents is on the basis of NTP (the National Toxicology Program) data set (Table 3). According to the results, 9 compounds were predicted to be non-toxic in development. All in all, compound 1 (ZINC000008860530) and compound 2(ZINC000004096987) were favorable agonists of DAR. Compound 1 and compound 2 didn’t have inhibition of CYP2D6’s activities and were non-hepatotoxic. Furthermore, they hardly had rodent carcinogenic, Ames mutagenic and developmental toxicity compared to others. Thus, the selected compounds are potential targeted drugs for DAR. As Figure 1 shown, the two selected compounds and Bromocriptine had similarities in their chemical structures. For example, all of them included several dual-band, multiple reactive oxygens and axisymmetric structure. What’s more, compound 1, compound 2 and Bromocriptine combined with DAR at the same region as well as position, close to DAR's G protein coupled receptor family. In summary, these two compounds had favorable safety. Therefore, they were selected as potential candidate compounds for further research (Figure 2).

**Table 3:** Toxicities of compounds.

**Number**	**Compounds**	**AMES^b^**	**DTP^c^**	**Mouse NTP^a^**			**Rat NTP^a^**	
				**Female**	**Male**		**Female**	**Male**
1	ZINC000004424205	0.104	0.838	0.364	0.786		0.301	0.294
2	ZINC000045337516	0.000	0.576	0.458	0.597		0.312	0.620
3	ZINC000014811803	0.011	0.772	0.289	0.540		0.247	0.488
4	ZINC000100590636	0.000	0.524	0.534	0.518		0.142	0.297
5	ZINC000017596232	0.000	0.397	0.265	0.025		0.241	0.232
6	ZINC000015122022	0.000	0.812	0.300	0.576		0.120	0.156
7	ZINC000100634117	0.000	0.524	0.534	0.518		0.142	0.297
8	ZINC000002566164	0.002	0.856	0.470	0.348		0.325	0.486
9	ZINC000100634116	0.000	0.524	0.534	0.518		0.142	0.297
10	ZINC000004097774	0.344	0.883	0.152	0.698		0.212	0.116
11	ZINC000002526388	0.000	0.618	0.299	0.439		0.422	0.483
12	ZINC000002528509	0.000	0.618	0.299	0.439		0.422	0.483
13	ZINC000008214547	0.000	0.957	0.851	0.924		0.261	0.395
14	ZINC000062238181	0.004	0.598	0.540	0.585		0.202	0.441
15	ZINC000008860530	0.000	0.361	0.636	0.614		0.145	0.306
16	ZINC000049180748	0.001	0.863	0.219	0.320		0.294	0.437
17	ZINC000004096987	0.006	0.282	0.529	0.487		0.159	0.368
18	ZINC000008221074	0.000	0.468	0.485	0.582		0.240	0.442
19	ZINC000011616465	0.000	0.329	0.123	0.021		0.071	0.541
20	ZINC000006920421	0.131	0.542	0.535	0.614		0.354	0.464
21	Bromocriptine	0.001	0.040	0.536	0.134		0.249	0.557

^a^<0.3 (Non-Carcinogen); >0.7 (Carcinogen).

^b^<0.3 (Non-Mutagen); >0.7 (Mutagen).

^c^<0.3 (Non-Toxic); >0.7 (Toxic).

### Ligand’s analysis binding and pharmacophore

**Figure 3:**
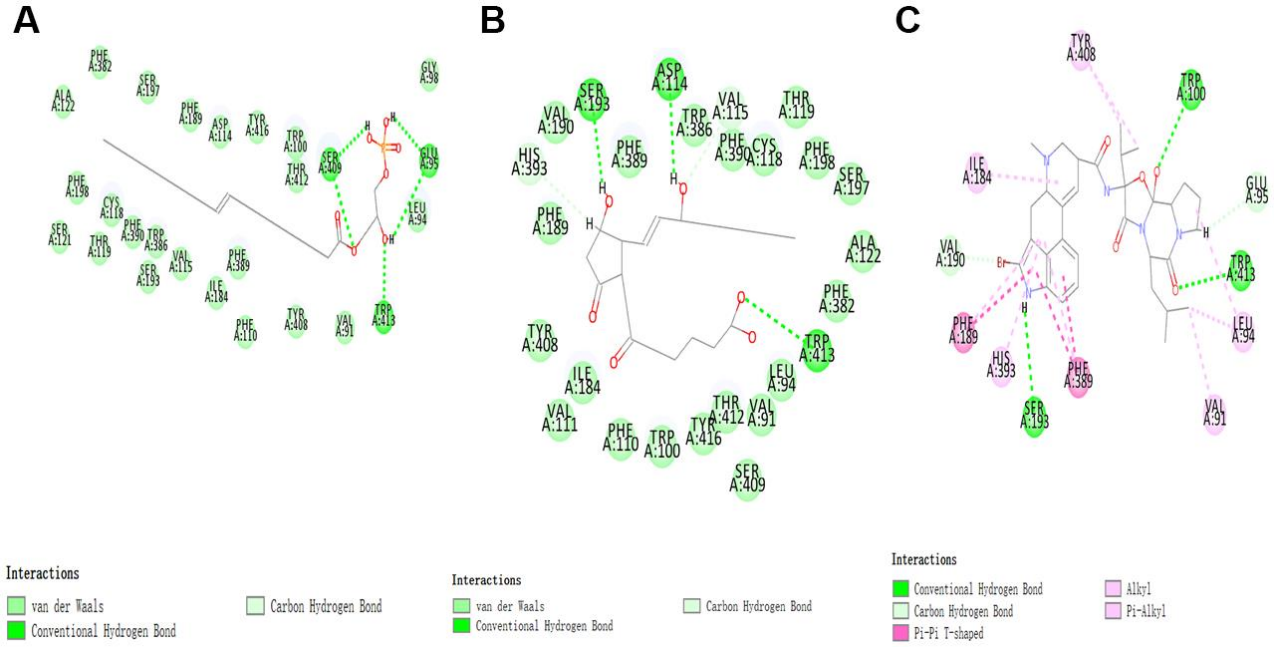
The inter-molecular interaction of the predicted binding modes of (**A**) ZINC000008860530 to Dopamine D2 Receptor; (**B**) ZINC000004096987 to Dopamine D2 Receptor, (**C**) Bromocriptine to Dopamine D2 Receptor.

**Figure 4:**
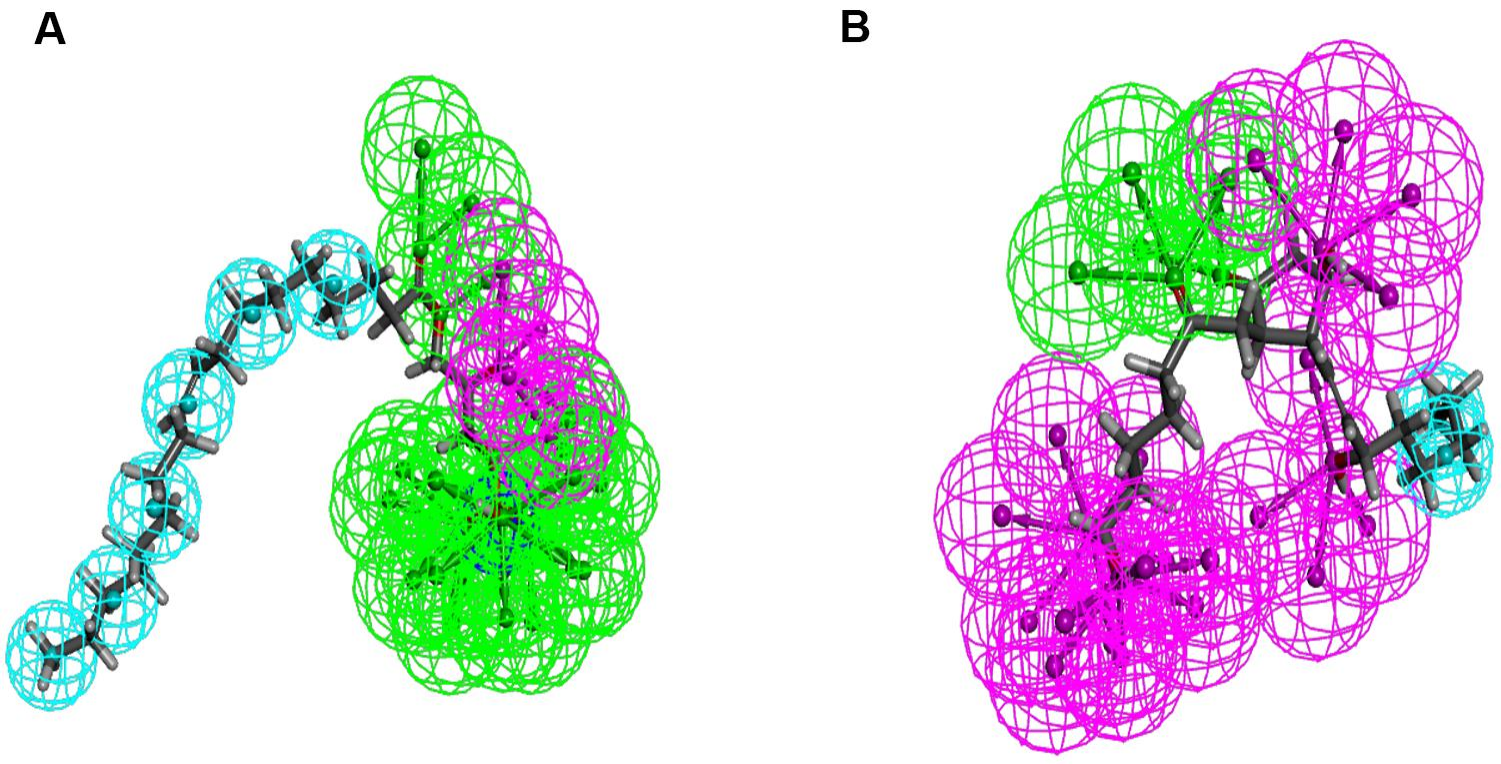
**Pharmacophore predictions using 3D-QSAR.** (**A**) ZINC000008860530: Green represents hydrogen acceptor, and blue represents hydrophobic center and purple represents hydrogen donor. (**B**) ZINC000004096987: Green represents hydrogen acceptor, blue represents hydrophobic center and purple represents hydrogen donor.

According to the results, the Root Mean Square Deviation (RMSD) was 0.6Å. It was between the complex’s crystal structure and the docked pose [22]. It indicated that CDOCKER module was very believable in this study. And CHARMm36 force field was applied to CDOCKER module. Two selected compounds were docked into DAR through CDOCKER module [23]. Table 4 showed that the CDOCKER Interaction energy of compound 1-DAR complex and compound 2-DAR complex was -63.9612 Kcal/mol and -57.1971 Kcal/mol separately. Nevertheless, Bromocriptine can't be docked into DAR this step. As we known, CDOCKER is a precise docking method under CHARMm forcefield. According to the result, the complex molecular structure of Bromocriptine is not conducive to accurate docking, which also shows that the two small molecules we selected can bind to the DAR protein more easily and stably. Additionally, to better evaluate the affinity and stability of the binding of molecules and proteins, we have also evaluated the absolute energy of protein and small molecule compounds. As we can see, Bromocriptine has higher absolute energy (143.83Kcal/mol) of compounds with Dopamine D2 Receptor than the compounds 1, 2. So, these two selected compounds might have higher affinity and stability when binding with DAR than Bromocriptine. Structures of ligands and DAR were also analyzed, including hydrogen bonds, Pi-Pi interaction, Alkyl interaction and Pi-Alkyl interaction [24]. As shown in Figures 2, 3 and Tables 5, 6, compound 1 and compound 2 didn’t form any Pi-Pi interaction, Alkyl interaction and Pi-Alkyl interaction. They formed several hydrogen bonds with DAR in the complexes. There were five hydrogen bonds formed by Compound 1 and DAR (A:GLU95:OE1-ZINC000008860530:H66, A:GLU95:OE1-ZINC000008860530:H69, A: TRP413:HE1-ZINC000008860530:O23, A:SER409:HG-ZINC000008860530:O20, A:SER409:OG-ZINC000008860530:H70). Similarly, three hydrogen bonds were formed by compound 2 and DAR (A:SER193:OG-ZINC000004096987:H44, A:ASP114:OD2-ZINC000004096987:H39, A:TRP413:HE1-ZINC000004096987:O26). Moreover, Bromocriptine created three hydrogen bonds with DAR, (A:SER193:OG-Bromocriptine:H68, A:TPR100:HE1-Bromocriptine:O14, A:TPR413:HE1-Bromocriptine:O7 independently). Additionally, there were three pairs of Pi-Pi interaction, four pairs of Alkyl interaction and five pairs of Pi-Alkyl interaction formed by Bromocriptine and DAR. And both ZINC000008860530 and ZINC000004096987 displayed several hydrogen bond acceptors, hydrophobic centres and hydrogen donors [25] (Figure 4). Computation results showed 22 feature pharmacophores in ZINC000008860530 and 29 feature pharmacophores in ZINC000004096987.

**Table 4:** CDOCKER interaction energy, relative energy and absolute energy of compounds with dopamine D2 receptor.

**Compounds**	**CDOCKER interaction energy (Kcal/mol)**	**Absolute energy (Kcal/mol)**
ZINC000008860530	-63.9612	32.9236
ZINC000004096987	-57.1971	33.3654
Bromocriptine	-	143.83

**Table 5:** Hydrogen bond interaction parameters for each compound and dopamine D2 receptor residues.

**Receptor**	**Compound**	**Donor atom**	**Receptor atom**	**Distances (Å)**
Dopamine D2 Receptor	ZINC000008860530	A:GLU95:OE1	ZINC000008860530:H66	2.92
		A:GLU95:OE1	ZINC000008860530:H69	1.99
		A:TRP413:HE1	ZINC000008860530:O23	2.55
		A:SER409:HG	ZINC000008860530:O20	2.57
		A:SER409:OG	ZINC000008860530:H70	2.58
	ZINC000004096987	A:SER193:OG	ZINC000004096987:H44	2.18
		A:ASP114:OD2	ZINC000004096987:H39	2.60
		A:TRP413:HE1	ZINC000004096987:O26	2.66
	Bromocriptine	A:SER193:OG	Bromocriptine:H68	2.84
		A:TPR100:HE1	Bromocriptine:O14	2.69
		A:TPR413:HE1	Bromocriptine:O7	2.23

**Table 6:** Pi-S interaction, Alkyl interaction and Pi-Alkyl interaction parameters for each compound and dopamine D2 receptor residues.

**Interaction parameters**	**Receptor**	**Compound**	**Donor atom**	**Receptor atom**	**Distances (Å)**
Pi-Pi interaction	Dopamine D2 Receptor	Bromocriptine	A:PHE189	Bromocriptine	5.21
			A:PHE389	Bromocriptine	4.49
			A:PHE389'	Bromocriptine'	4.3
Alkyl interaction		Bromocriptine	A:ILE184	Bromocriptine	5.08
			A:LEU94	Bromocriptine:C3	3.47
			A:LEU94	Bromocriptine	4.37
			A:VAL91	Bromocriptine:C3	3.36
Pi-Alkyl interaction		Bromocriptine	A:PHE189	Bromocriptine	5.35
			A:HIS393	Bromocriptine	4.83
			A:PHE389	Bromocriptine	4.5
			A:TYR408	Bromocriptine:C40	4.38
			A:TYR408	Bromocriptine:C39	3.46

**Figure 5:**
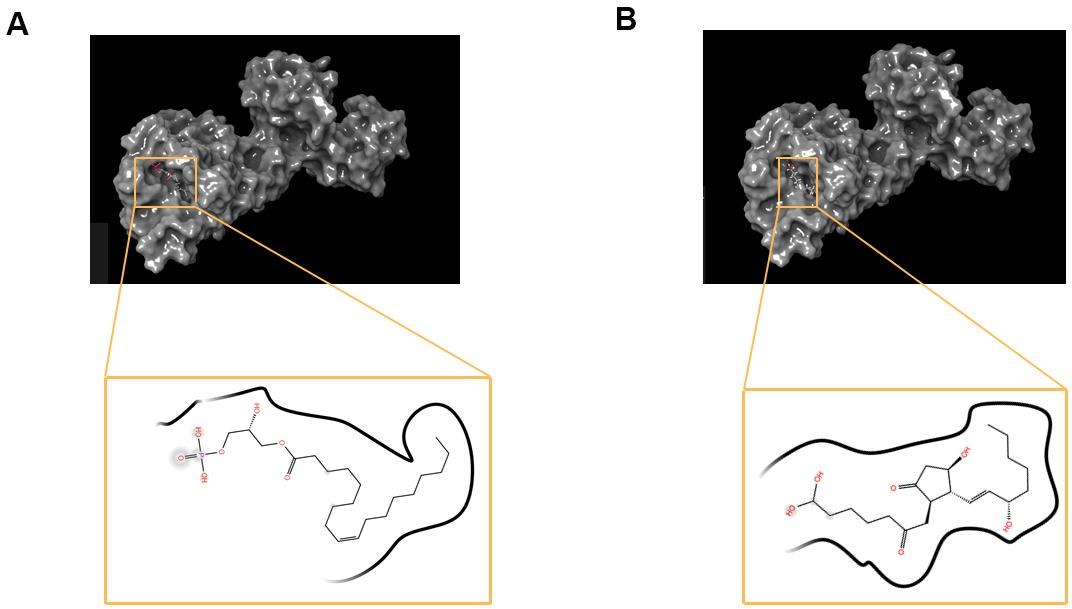
**The molecular docking by schrodinger.** Ligands were docked into the defined binding pocket. (**A**) ZINC000008860530 to Dopamine D2 Receptor. (**B**) ZINC000004096987 to Dopamine D2 Receptor.

**Figure 6:**
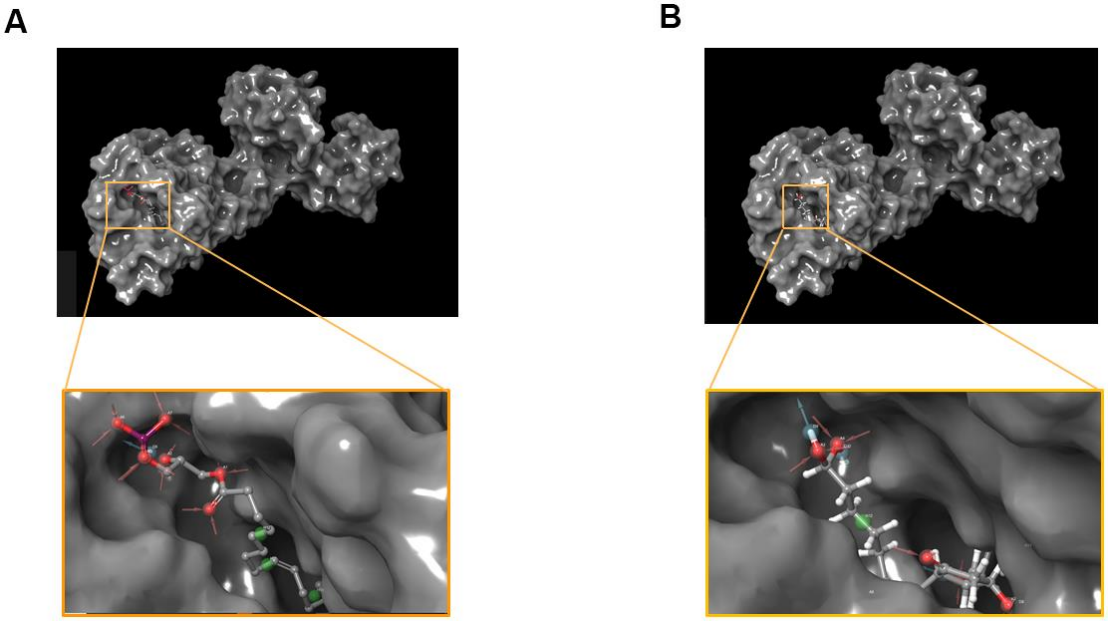
**Pharmacophore predictions using schrodinger.** (**A**) ZINC000008860530 to Dopamine D2 Receptor. (**B**) ZINC000004096987 to Dopamine D2 Receptor.

Additionally, to ensure the credibility of the results carried out with CDOCKER, the results were cross-checked again through Schrodinger. All docking conformations were visualized in order to ensure the docking at the designated place. The structures of compound 1-DAR complex and compound 2-DAR complex are shown in Figure 5. The pharmacophore part of result has also been supplemented by Schrodinger, such as the pharmacophore of small molecules in the docking conformation with the protein (Figure 6).

### Molecular dynamics simulation

**Figure 7:**
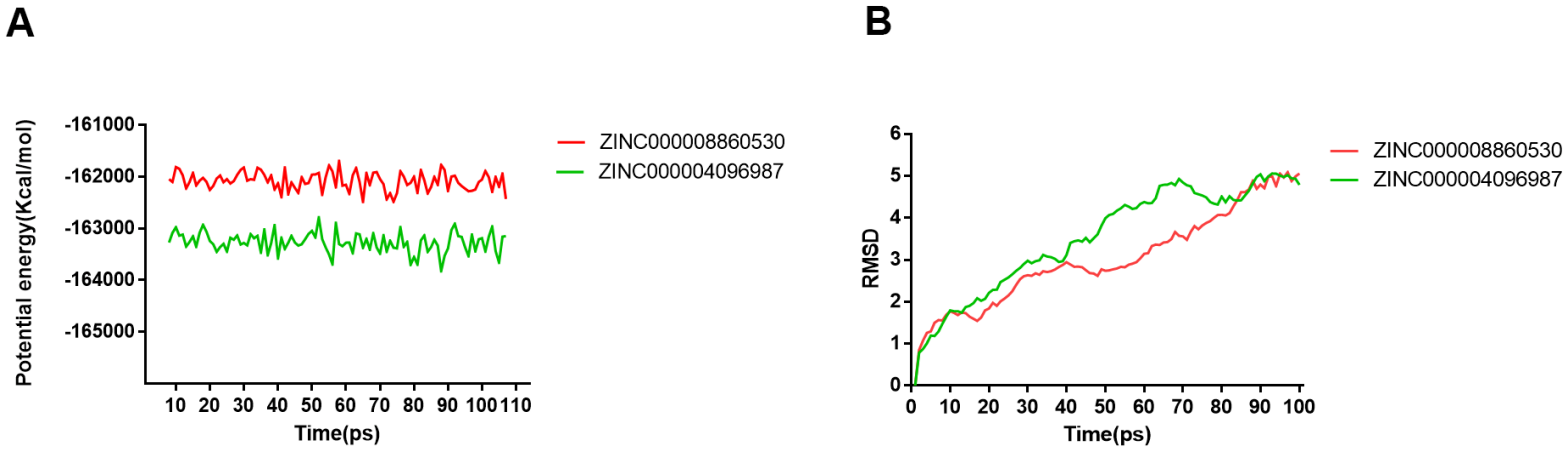
**Results of molecular dynamics simulation of two complexes.** (**A**) Potential Energy; (**B**) Average backbone RMSD.

Molecular dynamics simulation was performed to analyze and assess if ligand-DAR complex has excellent stability under the environment of nature. The results consisted of potential energy as well as RMSD curves profiles (Figure 7). It was at about 85 ps that these complexes’ RMSD curves got equilibrium. Additionally, these complexes’ RMSD and potential energy gradually got stable. According to the results, hydrogen bonds offered great promotion to the stability between compounds and DAR. So, under the environment of nature, compound 1, 2 have favorable stability and remarkable promotional effects binding to DAR than Bromocriptine did.

### Experiment to verify the therapeutic effect of compound 1, 2 on the viability of MMQ cells and PRL expression in MMQ cells

**Figure 8:**
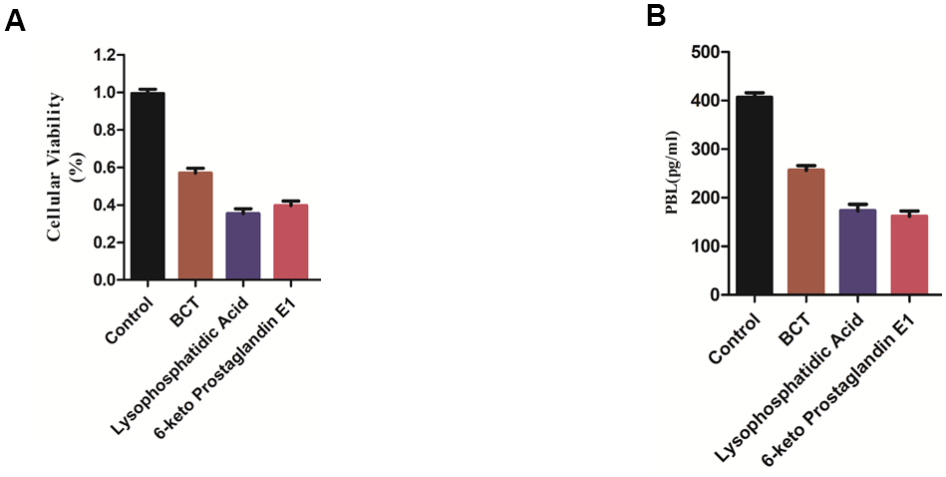
(**A**) Cellular viability of MMQ cells. (**B**) PRL expression in MMQ cells.

MMQ cells were treated with Bromocriptine, Lysophosphatidic Acid (ZINC000008860530) and 6-keto Prostaglandin E1(ZINC000004096987) for 72 hours. Then the cell viability was detected by CCK8 kit. The results showed that Bromocriptine, Lysophosphatidic Acid and 6-keto Prostaglandin E1 inhibited the MMQ cells’ proliferation compared with the blank control group. The cell viability of Bromocriptine, Lysophosphatidic Acid and 6-keto Prostaglandin E1 group was smaller than that of blank group, and the inhibitory effect of Lysophosphatidic Acid and 6-keto Prostaglandin E1 group on the proliferation of MMQ cells was stronger than that of Bromocriptine group (Figure 8A).

Additionally, the level of PRL secretion in, Bromocriptine, Lysophosphatidic Acid and 6-keto Prostaglandin E1 group was found lower than that in blank group, and the level of PRL secretion in Lysophosphatidic Acid and 6-keto Prostaglandin E1 group was lower than that in Bromocriptine group. It is suggested that Bromocriptine, Lysophosphatidic Acid and 6-keto Prostaglandin E1 group has an inhibitory effect on the level of PRL secretion of MMQ cells compared with the blank group (Figure 8B).

## DISCUSSION

DAR, a polypeptide chain consisting of 7 hydrophobic cross-membrane regions, is a new and vital drug target. Targeting dopamine receptors, DAR agonists are a class of drugs similar in function and structure to dopamine and can directly act on dopamine receptors [2, 3]. Clinically, DAR agonist drugs have achieved significant therapeutic effects on many diseases, such as prolactinoma, Parkinson's Disease, etc. [4].

Additionally, DAR includes d1, d2, d3, d4, d5 receptor subtypes [5]. And according to amino acid sequence and the signal conduction coupling, DAR is divided into D1 receptors (include d1 and d5 receptors subtypes) and D2 receptors (include d2, d3, and d4 receptor subtypes) [8, 26]. Furthermore, DAR agonists have different affinities, for different types of dopamine receptors, and they have different reactions and effects when acting on different dopamine receptors. And different dopamine receptors have different cell signaling pathway mechanisms. First of all, DAR couples with adenylate cyclase G protein (Gs) [27]. And by stimulating adenylate cyclase G protein (Gs), different DAR can positively or negatively couple with the Adenylate cyclase. D2 can activate Adenylate cyclase and increase the cAMP level in the cell, while D1 is the opposite [6]. In the cell signaling pathway, cAMP, as the second messenger, turns the inactive protein kinases into active, thereby activating phosphorylase and then causing reactions of target cells, such as glandular cell secretion, muscle cell contraction and relaxation, nerve cell potential changes, cellular Changes in permeability, cell division and differentiation, and various enzyme reactions, etc. [7].

Moreover, by acting on D2 receptors, DAR agonists have been applied to the clinical therapy of prolactinoma and Parkinson's disease for a long time and have an excellent therapeutic effect clinically [9]. Firstly, prolactinoma is a neuroendocrine-related disease characterized by excessive prolactin (PRL) secretion. And DAR agonists just happen to inhibit the secretion of PRL by binding to D2 DAR expressed on the surface of prolactinoma cells. So, DAR agonists benefit a lot in prolactinoma’ therapy. What’s more, agonists of DAR also have an ideal therapeutic effect on Parkinson's disease by regulating the dopamine pathway. Under normal circumstances, the dopamine pathway can be divided into two: direct pathway (D1 receptor participation) and indirect pathway (D2 receptor participation). Since Parkinson's disease is lack of dopamine, the effect on the direct pathway is weakened, and normal activities are reduced; the inhibitory effect on the indirect pathway is weakened so that the indirect pathway excessively inhibits unwanted exercise activities, resulting in symptoms such as reduced exercise and muscle stiffness [28]. Currently, DAR agonists on the market directly stimulate D2 receptors and have a weak inhibitory effect on D1 receptors so that the direct pathway and the indirect pathway return to normal or close to normal. The study thus plays a therapeutic role.

It can be seen that agonists of DAR have good prospects in the therapy of prolactinoma and Parkinson's Disease. Therefore, DAR agonists are a potential target drug molecule [14]. Nevertheless, many existing agonist-drugs of DAR have side effects, such as nausea, vomiting, headache, dizziness, etc. And there are fewer agonists of DAR with ideal therapeutic effect. Thus, studying the pharmacological effects of DAR agonists, finding drugs with high efficiency and low side effects, and applying them more widely in clinical practice, especially for prolactinoma and Parkinson’s disease, are very necessary and beneficial [29]. Among DAR agonists, Bromocriptine has been used in the clinical treatment of prolactin adenoma for the longest time and has an excellent therapeutic effect on prolactinoma and Parkinson's disease clinically [9]. Therefore, Bromocriptine was chosen as the reference molecule of DAR agonists in our study.

As for the research, several modules of DS 4.5 were applied to discover favorable DAR agonists as well as their analysis of pharmacology and toxicology. In addition, molecular conformation, the stability and affinity of binding were obtained, too. We downloaded a total of 25,932 small molecules from the ZINC15 database. The higher the LibDock score, the more stable the conformation of the compound and the more optimized the energy. According to LibDock’s results, 1429 compounds were proved to have appropriate binding with DAR. Moreover, Bromocriptine ranked 148th (147.011). So, these 147 compounds had better affinity and stability than Bromocriptine. Then the top 20 small molecules were selected for follow-up research.

Next, we studied the pharmacological properties and toxicological properties of small molecules. The results showed that compound 1 and compound 2 were the most favorable DAR agonists. First of all, their water solubility and intestinal absorption levels were ideal. Additionally, they would not inhibit CYP2D6(cytochrome P450 2D6)’s activities. And they were also not hepatotoxic. Moreover, the developmental toxicity, Ames mutagenicity and rodent carcinogenicity of these two selected compounds were relatively low. Therefore, these two small molecules were favorable DAR receptor agonists. In addition, regarding some compounds not selected in this study, they could be structurally modified to improve pharmacological properties and reduce toxicity. Therefore, they also had great value and prospects in the research and development of drug. In summary, compounds 1 and 2 are ideal DAR agonists. Therefore, we conducted further research on them. In addition, we analyzed the combination of these two compounds and DAR.

The CDOCKER interaction energy is an index for evaluating affinity. The lower it is, the higher the affinity between the compound and DAR. As the results (Table 4) shown, the CDOCKER Interaction energy compound 1-DAR complex and compound 2-DAR complex was -63.9612 Kcal/mol and -57.1971 Kcal/mol separately. Nevertheless, Bromocriptine can't be docked into DAR this step. As we have known, CDOCKER is based on the CHARMm forcefield, which is a more accurate molecular docking method than LibDock. According to the result, the complex molecular structure of Bromocriptine is not conducive to accurate docking, which also shows that the two small molecules we selected can bind to the DAR protein more easily and stably. Additionally, in order to better evaluate the affinity and stability of the binding of molecules and proteins, we have also assessed the absolute energy of protein and small molecule compounds. As we can see, Bromocriptine has higher absolute energy (143.83Kcal/mol) of compounds with Dopamine D2 Receptor than compounds 1, 2. Therefore, the binding affinity and stability of the two selected compounds to DAR might be higher than bromocriptine. In addition, compounds 1, 2 and bromocriptine have similar chemical structures, such as several multiple reactive oxygen compositions and double bands. And we found that the chemical structure of compound 1 (ZINC000008860530), compound 2 (ZINC000004096987) and bromocriptine had many similarities. For example, all of them has an axisymmetric structure. Moreover, position and distinct where these two potential compounds and Bromocriptine both combine with DAR are same. To summarize, these two compounds have good safety. So, they are chosen for next study.

In the calculation and prediction of pharmacophores, these two compounds might have appropriate effective pharmacophores. ZINC000008860530 had 22 feature pharmacophores, including hydrogen bond acceptors, hydrophobic centers and characteristic pharmacophores for hydrogen donors. ZINC000004096987 had 29 feature pharmacophores, including hydrogen bond acceptors, hydrogen bond donors, and characteristic pharmacophores for hydrophobic centers. In subsequent drug research, functional groups can be added to these two candidate compounds to improve efficacy and reduce side effects.

Additionally, in order to make the results more credible, we verified the results of CDOCKER with Schrodinger. The results show that the docking region of the ligands and protein were a defined binding pocket. And the pharmacophore part of result supplemented by Schrodinger were also shown in the docking conformation with the protein.

Next, we performed molecular dynamics simulation analysis. In this procedure, we aimed to assess the stability of the binding of small molecules to DAR. The RMSD and the potential energy curves of these complexes gradually stabilized and reached equilibrium at about 85 ps. Therefore, compound 1, compound 1 and DAR’s complexes will have significant effects and remain stable similar to bromocriptine in natural environments.

Through the above molecular simulation docking, we found two potential DAR agonists. All of their docking indicators are better than the control drug, Bromocriptine (a recognized dopamine receptor agonist). As far as we know, dopamine receptor agonists, such as Bromocriptine, are effective in the treatment of prolactinoma. Therefore, we predict that they can be as effective as the control Bromocriptine in the treatment of prolactinoma and even more effective than Bromocriptine. Then, CCK8 assay and ELISA *in vitro* were performed to assess the effects of potential DAR agonists in the study. As we all know, prolactinoma is the most common type of pituitary tumors which is characterized by excessive prolactin (PRL) secretion as the neuroendocrine-related disease. Currently, it is believed that DAR agonists can inhibit the secretion of PRL by binding to D2 DAR expressed on the surface of normal prolactin and pituitary tumor cells13. Moreover, D2 DAR are activated to inhibit prolactinoma cell proliferation and reduce cell volume [13]. We chose the secretion level of prolactin (PRL) and the proliferation level of prolactinoma cell as the evaluation indicators to assess the drug effect. In ELISA assay, the level of PRL secretion in Lysophosphatidic Acid and 6-keto Prostaglandin E1 group was lower than that in Bromocriptine group. In CCK8 assay, the cellular viability in cell lines MMQ treated with Lysophosphatidic Acid and 6-keto Prostaglandin E1 was smaller than that of Bromocriptine. Therefore, the results demonstrated that the effect of Lysophosphatidic Acid and 6-keto Prostaglandin E1 was better that of Bromocriptine in anti- pituitary tumors.

All in all, we are trying to find more suitable and effective DAR agonist drugs. We first carried out computer simulation screening, and then carried out experiments to confirm our results. The compounds identified in this study can greatly promote the development of drugs for prolactinoma and Parkinson's disease. Although this research was thoroughly arranged and designed, there are still some limitations. The safety of the drug needs further animal experiments and cell experiments to verify it. If necessary, some necessary groups can be added to reduce the toxicity of the drug. This is also the direction our laboratory is working hard to study.

## CONCLUSIONS

We applied computer simulation analysis and structural biology methods to screen out ideal DAR agonists. Subsequent experiments were also conducted to verify the efficacy of the drug. In addition, toxicological analysis of these two small molecules showed that their toxicity was within a reasonable range. In short, both compounds 1 and 2 are ideal potential agonists of DAR. This study promote the development of drugs for prolactinoma and Parkinson's disease.

## MATERIALS AND METHODS

### Software for docking and molecular database

Discovery Studio 4.5 is a comprehensive molecular modeling and environmental simulation software launched by biovia. It has chemical/biological data display, simulation/analysis, construction of three-dimensional molecules, display of dynamic changes, three-dimensional mapping and many other functions. In addition, this software is commonly used in small molecule drug screening. Firstly, LibDock was used for preliminary screening. Absorption, distribution, metabolism and excretion (ADME) were assessed to analyze the pharmacological properties of small molecules. Additionally, CDOCKER was performed to dock molecules with DAR more precisely. In the research, we got DAR agonists from the ZINC15 database. ZINC15 database is a database free of commercially-available database. This database is offered by UCSF (the Irwin and Shoichet Laboratories in the Department of Pharmaceutical Chemistry at the University of California, San Francisco).

### Virtual screening by LibDock

**Figure 9:**
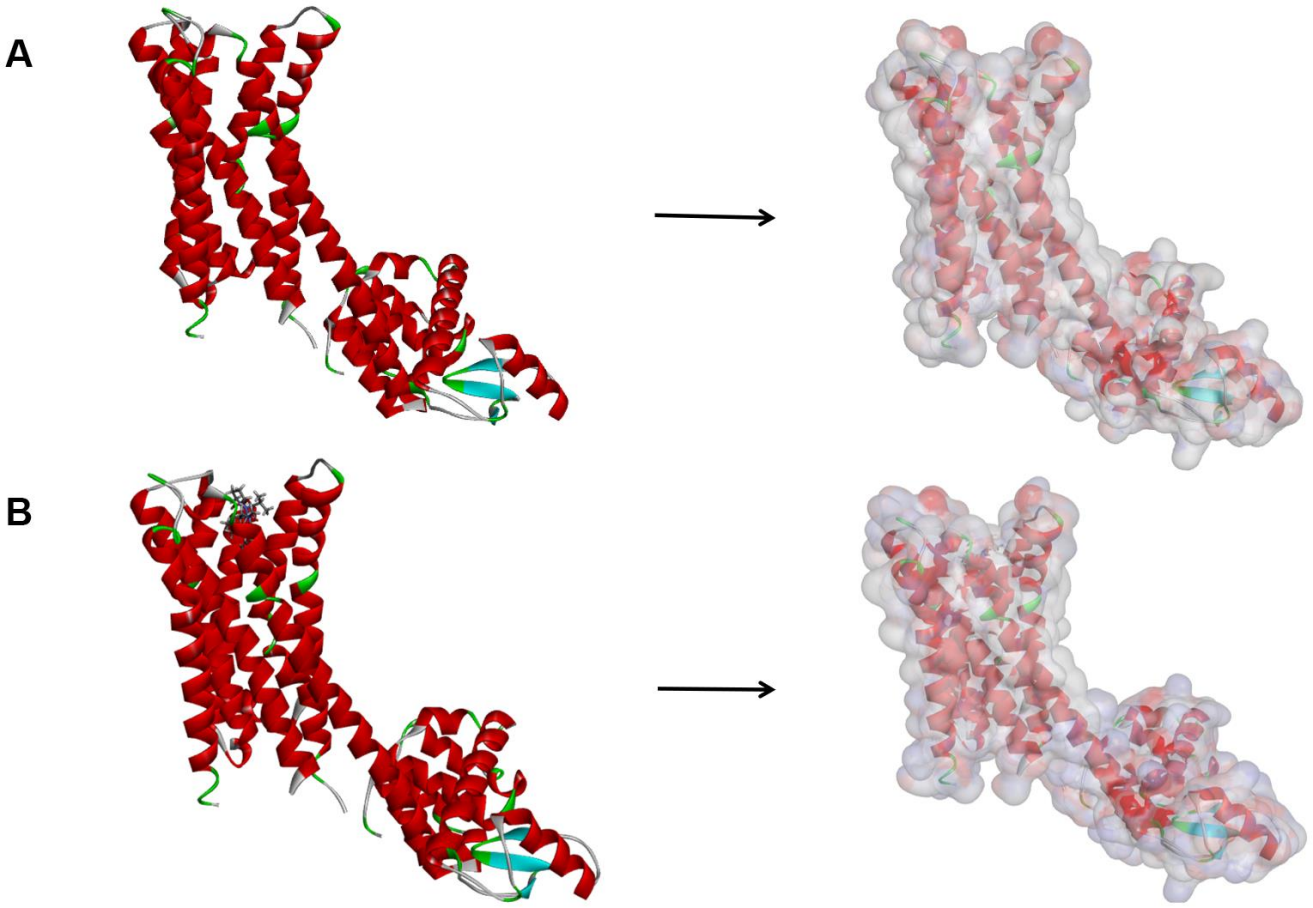
(**A**) The molecular structure of Dopamine D2 Receptor. Initial molecular structure was shown, and the surface of the molecule was added. (**B**) The complex structure of Dopamine D2 Receptor with Bromocriptine. Initial complex structure was shown, and the surface of the complex was added. Blue represented positive charge, red represented negative charge.

Firstly, we located the region where the DAR’s ligand Bromocriptine and DAR combine with each other. Then the sphere binding site was chosen for virtual screening of the potential favorable ligands. LibDock is a program based on rigid docking. Complex’s hotspots were analyzed and assessed and then applied to help and coordinate the molecules to bind appropriately. Next, all ligands with different poses are ranked according to the LibDock scores. We got DAR's structure with Bromocriptine (Parkinson's Disease B ID: 6CM4, 2.55Å) from the Protein Database (PDB), followed by LibDock analysis. Chemical structure of DAR was shown in Figure 9. Then, water and other heteroatoms were first removed from protein in the protein modification stage. Hydrogen, protonation, ionization all were added and energy minimization was performed next. In addition, the minimization of molecule was performed based on the the Smart Minimiser algorithm and CHARMm force field. Moreover, we set the RMS (root mean square) to 0.1 and the RMS gradient to 0.09778 to minimize the execution of 2000 steps. Afterwards, the combined ball's parameters were set, and the binding region of DAR's ligand Bromocriptine is chosen as an ideal docking site. Then, libdock was applied to virtually screen potential favorable ligands by docking them to defined sphere binding site. Afterwards, all ligands with different poses were ranked by the scores of LibDock.

### ADME and toxicity prediction

The ADME (absorption, distribution, metabolism and excretion) module of DS 4.5 is applied for the analysis and assessment of water-solubility, Cytochrome P450 2D6 (CYP2D6) inhibition, toxicity, plasma protein binding (PPB) levels, human intestinal tract Absorption, hepatotoxicity and blood-brain barrier (BBB) penetration of the selected favorable compounds. Besides, TOPKAT is the analysis that toxicity is predicted by Komputer Assisted Technology. And TOPKAT is utilized to analyze and predict molecules’ toxicities, such as Ames Mutagenicity, developmental toxicity potential, rodent carcinogenicity. Pharmacological properties predicted by ADME and Toxicity analysis will be considered when a potential candidate ligand of DAR agonist is chosen.

### More precise molecular docking and pharmacological analysis

CDOCKER docking was performed under the CHARMm36 forcefield, which was more accurate than Libdock docking. During the docking process, the structure of the protein receptor remained rigid, while the structure of the ligand was very flexible. After the docking was completed, we analyzed the interaction energy and CHARMm energy of each complex in various postures. These two energies were indicators that reflected the binding affinity of the ligand. Firstly, we downloaded the crystal structure of DAR from the PDB protein database. Afterwards, we used Discovery Studio 4.5 software to process and prepare the protein to make it meet the docking conditions. Moreover, since crystalline water molecules and water molecules may affect the binding of receptors and ligands during docking procedure, they were deleted. Also, polar hydrogen atoms were added to DAR and CHARMm36 forcefield were applied to the DAR and ligands. In addition, we deleted the bromocriptine molecule from the protein, and then re-docked it to DAR to make it more believable. The binding site was set as the binding sphere within a radius of 16Å centered on the binding site of bromocriptine and DAR. Each ligand recognized, interacted and bound to the residues in the receptor binding sphere. Finally, the CDOCKER interaction energy of different docking poses of every ligand would be displayed. We choose CDOCKER molecules with higher interaction energy poses for follow-up research. Furthermore, we also used the 3D-QSAR pharmacophore generation module was also carried out. And the pharmacophore of the compound was displayed. Each molecule can generate up to 255 confirmations. But, only those with energy lower than 10 kcal/mol could be retained.

Additionally, to make the results more credible carried out by CDOCKER, the procedure were crosschecked again with Schrodinger. And the pharmacophore of small molecules in the docking conformation with the protein was performed by Schrodinger.

### Molecular dynamics simulation

We selected the best conformation of compound 1, compound 2, Bromocriptine and DAR for this step of molecular dynamics simulation. Firstly, we put the complex of compound 1, compound 2, Bromocriptine and DAR into an orthorhombic box. Next, we solvated it with an explicit periodic boundary solvated water model. In addition, to analyze the physiological environment, solid chloride with an ionic strength of 0.145 were added. Moreover, we assigned a CHARMm forcefield to the system. After that, we relaxed the complex by minimizing the energy (500 steps for steepest descent and 500 steps for conjugate gradient). The final RMS gradient was 0.289. Under the equilibrium simulation of 200 ps and 250 ps, the system temperature increased from 50 K to 300 K. The time step was set to 2fs. The process was carried out on an NPT (atmospheric pressure and temperature) system. And temperature was set at a constant temperature of 300 K. We also used the PME (particle grid Ewald) algorithm to analyze remote static electricity, and adjusted the LINCS (Linear Constraint Solver) algorithm accordingly to make all hydrogen-related bonds fixed [18]. With reference to the initial settings, we performed the trajectory protocol steps in DS 4.5 to analyze the structural performance, RMSD and potential energy, and drew trajectory [18].

### Experiment to verify the therapeutic effect of compound 1 and compound 2 on the viability of MMQ cells and PRL expression in MMQ cells

### Experimental reagents and supplies

MMQ cell lines (Shanghai Zeye Biological Technology Co., Ltd.); ELISA detection kit (bought from GE Healthcare); DMEM high glucose medium (bought from Gibco); Bromocriptine (bought from BCT); Lysophosphatidic Acid (ZINC000008860530) and 6-keto Prostaglandin E1(ZINC000004096987) (bought from Santa Cruz Animal Health Company); other experimental reagents (bought from Sigma).

### Cell culture

The culture conditions of the MMQ cell line are high glucose DMEM medium with 10% fetal bovine serum, 37° C and 5% CO2. We only pass the cells once. In the logarithmic growth phase of the cells, the morphology of the cells was examined with an optical microscope.

### CCK8 assay

We seeded MMQ cells into 96-well plates at a density of 5×103/well, with 3 duplicate wells in each group. After 24h, Bromocriptine, Lysophosphatidic Acid and 6-keto Prostaglandin E1 were added into 96-well plates at a certain concentration and then cultured in 5%CO2 at 37° C for 72 h. Add 100 μL test solution (including 10 μL CCK8+90 μL DMEM medium) to each well and incubate at 37° C for 1h. The absorbance of the solution at 450 nm was determined by an enzyme plate analyzer.

### Detection of PRL

The cell culture and grouping were the same as mentioned above. According to the ELISA detection kit's instructions, the operation was carried out, and the PRL expression of MMQ cells was measured.

### Supporting information

Figure 2. The charge surface of the junction pocket was added, blue represented positive charge, red represented negative charge, and ligands were shown in the sticks, the structure around the ligand-receptor junction as shown in thinner sticks. (A) ZINC000008860530-DAR complex; (B) ZINC000004096987-DAR complex; (C) Bromocriptine-DAR complex.
